# Mesenchymal stem cells ameliorate myocardial fibrosis in diabetic cardiomyopathy via the secretion of prostaglandin E2

**DOI:** 10.1186/s13287-020-01633-7

**Published:** 2020-03-17

**Authors:** Liyuan Jin, Jinying Zhang, Zihui Deng, Jiejie Liu, Weidong Han, Guanghui Chen, Yiling Si, Ping Ye

**Affiliations:** 1 Department of Geriatric Cardiology, Chinese PLA General Hospital, No. 28, Fuxing Road, Beijing, 100853 China; 2 Chinese People’s Liberation Army Medical School, No. 28 Fuxing Road, Beijing, 100853 China; 3 Department of Basic Research, Chinese PLA General Hospital, No. 28 Fuxing Road, Beijing, 100853 China; 4 Department of Cardiology, Chinese PLA General Hospital, No. 28 Fuxing Road, Beijing, 100853 China

**Keywords:** Diabetic cardiomyopathy, Mesenchymal stem cell, Myocardial fibrosis, Prostaglandin E2

## Abstract

**Background:**

Diabetic cardiomyopathy (DCM) is a cardiac complication of long-term uncontrolled diabetes and is characterized by myocardial fibrosis and abnormal cardiac function. Mesenchymal stem cells (MSCs) are multipotent cells with immunoregulatory and secretory functions in diabetes and heart diseases. However, very few studies have focused on the effect and the underlying mechanism of MSCs on myocardial fibrosis in DCM. Therefore, we aimed to explore the therapeutic potential of MSCs in myocardial fibrosis and its underlying mechanism in vivo and in vitro.

**Methods:**

A DCM rat model was induced using a high-fat diet (HFD) combined with a low-dose streptozotocin (STZ) injection. After four infusions of MSCs, rat serum and heart tissues were collected, and the levels of blood glucose and lipid, cardiac structure, and function, and the degree of myocardial fibrosis including the expression levels of pro-fibrotic factor and collagen were analyzed using biochemical methods, echocardiography, histopathology, polymerase chain reaction (PCR), and enzyme-linked immunosorbent assay (ELISA). We infused prostaglandin E2 (PGE2)-deficient MSCs to DCM rats in vivo and established a system mimicking diabetic myocardial fibrosis in vitro by inducing cardiac fibroblasts with high glucose (HG) and coculturing them with MSCs or PGE2-deficient MSCs to further explore the underlying mechanism of amelioration of myocardial fibrosis by MSCs.

**Results:**

Metabolic abnormalities, myocardial fibrosis, and cardiac dysfunction in DCM rats were significantly ameliorated after treatment with MSCs. Moreover, the levels of TGF-β, collagen I, collagen III, and collagen accumulation were markedly decreased after MSC infusion compared to those in DCM hearts. However, PGE2-deficient MSCs had decreased ability to alleviate cardiac fibrosis and dysfunction. In addition, in vitro study revealed that the concentration of PGE2 in the MSC group was enhanced, while the proliferation and collagen secretion of cardiac fibroblasts were reduced after MSC treatment. However, MSCs had little effect on alleviating fibrosis when the fibroblasts were pretreated with cyclooxygenase-2 (COX-2) inhibitors, which also inhibited PGE2 secretion. This phenomenon could be reversed by adding PGE2.

**Conclusions:**

Our results indicated that MSC infusion could ameliorate cardiac fibrosis and dysfunction in DCM rats. The underlying mechanisms might involve the function of PGE2 secreted by MSCs.

## Background

Diabetes is one of the major chronic diseases and will affect approximately 450 million people worldwide by 2030. Cardiovascular complications, including diabetic cardiomyopathy (DCM), represent the leading causes of morbidity and mortality in diabetic patients [1, 2]. DCM is mainly characterized by myocardial fibrosis, chronic inflammation, and structural and functional cardiac changes caused by long-term glucose abnormalities [3–5]. In particular, cardiac fibrosis is often accompanied by increased pro-fibrotic factors such as transforming growth factor (TGF)-β, proliferation and dysfunction of cardiac fibroblasts, increased deposition, and decreased degradation of extracellular matrix proteins (ECM) such as collagen, leading to increased ventricular wall stiffness, abnormal cardiac filling, and diastolic and contractile cardiac dysfunction [6–8]. Finally, uncontrolled cardiac injury in DCM results in irreversible heart failure and death. Unfortunately, there is still no effective treatment to prevent the progression of heart failure.

More recent studies found that mesenchymal stem cells (MSCs) have important secretory, immunoregulatory and anti-inflammatory functions and therapeutic potential for diabetes and certain heart diseases [9, 10]. A few studies have demonstrated the beneficial effects of MSCs on DCM. However, there is limited knowledge regarding the potential mechanisms underlying the positive effects of MSCs on myocardial fibrosis in DCM. Based on prior studies and our previous research, MSCs might secrete cytokines, especially PGE2, to produce local and systemic effects [11–14]. In addition, it has been shown that PGE2 exhibits an anti-fibrotic effect in pulmonary and cardiac fibrotic diseases [15–17]. Therefore, the current research intended to study the potential mechanism underlying the effect of MSCs on cardiac fibrosis of an established DCM model in vivo and in vitro, aiming to provide theoretical support for clinical treatment of DCM.

## Materials and methods

### Animal experiments

#### Induction of DCM rat model

The induced type 2 diabetes rat model was established as previously described [13]. Eight-week-old male Sprague-Dawley (SD) rats were obtained from the Chinese People’s Liberation Army (PLA) General Hospital and divided into 3 groups including the normal group, DCM group, and MSCs group. The DCM and MSCs groups were fed a high-fat diet (HFD) (40% fat, 41% carbohydrate, and 19% protein) and the normal group was fed a normal chow diet for 8 weeks. Then, a single dose of 25 mg/kg streptozotocin (STZ, Sigma-Aldrich) was intraperitoneally injected into the HFD-fed rats. Four weeks after STZ injection, the DCM rat model was evaluated and confirmed by echocardiography. All protocols were approved by the medical ethics committee of the Chinese PLA General Hospital.

#### AD-MSCs administration

Twelve weeks after DCM rat model induction and evaluation, 2 × 10^6^ adipose-derived MSCs (AD-MSCs) or PGE2-deficient MSCs suspended in 0.3 mL of sterile physiological saline were infused into rats of the MSCs group or MSCs-PGE2^−/−^ group via tail vein every 2 weeks, 4 times in total. The normal and DCM groups were infused with 0.3 mL of sterile physiological saline as the control.

### Virus transfection in MSCs

#### Construction of PGE2 deficient MSCs

Passage 2 MSCs were transfected with Ptges-RNAi to knock down the expression of PGE2 in MSCs or negative lentivirus as control (GENECHEM, Shanghai, China). After 48 h, transfected cells with green fluorescence observed under a fluorescence microscope were cultivated with 4 μg/mL puromycin for another 4 days until no cell died anymore. Left cells were amplified, photographed under a fluorescence microscope and detected by ELISA.

#### Luciferase-labeled lentivirus transfected in MSCs

MSCs were transfected with Ubiquitin-firefly-Luciferase-labeled lentivirus (GENECHEM, Shanghai, China). After 96-h transfection, cells were screened under the 4 μg/mL puromycin for another 5 days until no cell died anymore. Then, the labeled cells were amplified.

### Biochemical and basal metabolic examinations

Rats were weighed throughout the experiment. Hearts were weighed, and the ratio of heart weight to body weight was calculated at the end of the experiment. Daily food and water intake was calculated throughout the experiment. Blood from the tail vein was collected at the beginning, 12 weeks, and 20 weeks. Biochemical examinations including fasting blood glucose (FBG), serum insulin (INS), total cholesterol (TC), and triglyceride (TG) levels were detected by ELISA or biochemical analyzer at the time points of 0 week, 12 weeks, and 20 weeks.

### Echocardiography

Two-dimensional, M-mode echocardiogram, pulsed-wave Doppler imaging, and tissue Doppler were performed using a Vevo 770 ultrasound system under anesthesia with isoflurane. Serial echocardiography was performed at the 12-week time point and at the end of the experiment, respectively. To determine cardiac structure, left ventricular end-diastolic internal diameters (LVIDD), LV end-systolic internal diameters (LVIDS), LV end-systolic anterior wall (LVAWS), and LV end-systolic posterior wall thickness (LVPWS) were measured by parasternal short and long-axis scans at the papillary muscle level. To assess cardiac systolic function, the fractional shortening (FS) was calculated simultaneously, and the left ventricular ejection fraction (LVEF) was measured by parasternal long-axis scans. In addition, pulsed-wave and tissue Doppler were used to measure the early to late diastolic transmitral flow velocity (E/A) ratio and the early to late diastolic mitral annular velocity (E’/A’) ratio to assess cardiac diastolic function.

### Histopathology staining and analysis

Rat hearts were isolated, imaged, weighed and measured after death at week 20. Hearts were fixed in formalin, embedded in paraffin, and cut into three 4-μm sections at the papillary muscle level. To determine myocardial fibrosis, heart sections were stained with Picrosirius red. Cardiac interstitial fibrosis was determined on the collagen fibers stained with red among cardiomyocytes or around vessels. The fibrosis was quantified by calculating the percentage of collagen staining with five randomly selected images in every section using ImageJ software analysis.

### Optical bioluminescence imaging

The luciferase-labeled MSCs were injected into rats via tail vein, and rats were injected substrate luciferin and imaged by optical bioluminescence (IVIS Spectrum, PerkinElmer) in vivo at 1 day, 1 week, and 2 weeks, respectively. Otherwise, organs containing lungs, heart, kidneys, brain, and liver were taken bioluminescence imaging ex vivo immediately after in vivo imaging. The images and data were analyzed with Living Image.

### Cell experiments

#### Isolation, cultivation, and identification of adipose-derived MSCs

Male Sprague-Dawley (SD) rats weighing 80–100 g were sacrificed by cervical dislocation, and then disinfected in 75% alcohol. Adipose-derived (AD)-MSCs were isolated from inguinal fat tissue as described previously [18, 19]. Briefly, adipose tissue was minced into 1 mm^3^ pieces with eye scissors, and the pieces were digested with 0.1% type I collagenase and 0.05% trypsin for 40–50 min at 37 °C, terminated with 10% fetal bovine serum (FBS), and centrifuged at 1500 rpm for 5 min. Sediment was suspended with Dulbecco’s modified Eagle’s medium with l-glutamine (DMEM-L) containing 10% FBS and filtrated through a 200-mesh strainer. The filtered liquid was centrifuged and suspended again. The cell suspension was cultivated at 37 °C with 5% CO_2_. After three passages, AD-MSCs were cultured in either an adipogenesis-inducing solution (Cyagen) for 2 weeks and stained with Oil Red O (Sigma) or an osteogenesis-inducing fluid (Cyagen) for 2 weeks and stained with Alizarin red (Sigma). In addition, AD-MSCs were expanded to passage three or four and identified by flow cytometry. AD-MSCs were stained with APC-conjugated CD29 (eBioscience), CD90 (BD) antibodies and FITC-conjugated CD34, CD45, and CD11b antibodies (BD).

#### Isolation, cultivation, and identification of cardiac fibroblasts

One-day-old SD neonatal rat cardiac fibroblasts were isolated, purified, and cultured as described before [20, 21]. In brief, SD neonatal rats were disinfected by 75% alcohol, and the heart was harvested by sterile forceps and put into cold phosphate-buffered saline (PBS) to wash off the blood. Then, large vessels and connective tissue were removed from the heart, minced into small pieces (1 mm^3^) with eye scissors, and cleaned with 0.08% trypsin (Hyclone) at 37 °C for 5 min. The initial digestive liquid was discarded, and the remaining pieces were digested with 0.08% type II collagenase (Gibco) at 37 °C for 5 min in a rotary incubator. The digestive liquid was terminated with 10% FBS medium until the tissue was dissolved completely. The digestion was filtered through a 200-mesh strainer, and the filtered liquid was centrifuged at 300×*g* for 10 min. The supernatant was discarded, and sediment was suspended with Dulbecco’s modified Eagle’s medium–high glucose (DMEM-HG; HyClone) containing 15% FBS, and the cell suspension was cultivated at 37 °C with 5% CO_2_ for 90 min to obtain fast adhering fibroblasts. Cardiac fibroblasts were cultivated with DMEM-H complete medium containing 10% FBS, 100 U/mL of penicillin, and 100 g/mL of streptomycin and expanded to 3 or 4 passages. Passage 3 fibroblasts were transplanted in a confocal dish, and immunofluorescent cell identification was performed with vimentin and cTnT antibodies until approximately 80–90% confluent.

#### In vitro experiments

Passage 3 cardiac fibroblasts were seeded into a 6-well culture plate, after a 6-h incubation, the culture medium was replaced with a high-glucose medium (33 mmol/L) and incubated for another 6 h. Then, AD-MSCs were cocultured with cardiac fibroblasts at a ratio of 1:2 (MSC:fibroblast) for 48 h in a Transwell system (Corning). Before that, AD-MSCs were seeded into the upper chamber of a Transwell culture plate for 24 h and pretreated with cyclooxygenase-2 (COX-2) inhibitor (10 μM, Abcam) for 2 h before coculturing with fibroblasts either with or without adding prostaglandin E2 (PGE2; 0.1 μM, Sigma) to the coculture system. The grouping was as follows: (1) normal, (2) normal + MSCs, (3) high glucose (HG), (4) HG + MSCs, (5) HG + MSCs + COX-2 inhibitor, and (6) HG + MSCs + COX-2 inhibitor + PGE2.

### Flow cytometry analysis

AD-MSCs were digested using 0.25% trypsin and terminated with 10% FBS, then centrifuged at 1500 rpm for 5 min. The pellet was suspended with cold PBS and washed twice. The cell pellet was suspended with 100 μL of PBS and incubated with antibodies against CD29 (APC, eBioscience), CD90 (APC, BD), CD34, CD45, and CD11b (FITC, BD) at room temperature for 20 min away from light. AD-MSCs were washed with PBS and analyzed using a FACSCalibur flow cytometer (BD Biosciences) with CellQuest software. Unlabelled cells were used as negative control.

### Immunofluorescence staining

Cardiac fibroblasts in the confocal dish were washed twice with PBS and fixed with 4% paraformaldehyde for 20 min. Cells were washed with PBS for 5 min and perforated by 0.5% TritonX-100 for 15 min. Then, cells were washed twice with PBS for 5 min each time and blocked with 20% goat serum at 37 °C for 30 min. After that, cells were incubated with diluted primary antibodies of vimentin (Bioss) and cTnT (Abcam) overnight at 4 °C. The next day, cells were washed twice and incubated with Alexa Fluor 488 and Alexa Fluor 555-conjugated secondary antibodies at room temperature for 1 h away from light and washed twice. Finally, cells were stained with 4, 6-diamidino-2-phenylindole (DAPI; Sigma-Aldrich) for 5 min and washed again. Cells were observed using an inverted fluorescence microscope (Olympus IX71, Japan) after mounting under glycerol.

### Cell counting kit-8 assay

Cardiac fibroblasts were seeded in a 24-well plate (approximately 1 × 10^5^ cells/well). After different treatment, cell proliferation was evaluated using cell counting kit-8 (CCK-8) assay (Dojindo, Kumamoto, Japan) according to the manufacturer’s instructions. The absorbance value of each well was determined at 450 nm using the instrument SYNERGY LX (SLXFA, America).

### Quantitative real-time reverse transcriptase polymerase chain reaction

Total RNA of hearts and cardiac fibroblasts were extracted using TRIzol reagent (Ltd RP1105, Solarbio, Beijing) and transcribed to cDNA with a reverse transcription kit. Real-time polymerase chain reaction (PCR) was performed with a SYBR Green PCR master mix (Ltd RP1110, Solarbio, Beijing) using Molarray (Ltd MA-6000, Suzhou). The primer sequences for TGF-β and glyceraldehyde 3-phosphate dehydrogenase (GAPDH) are listed in Supplemental Table 1. A melt curve was included to ensure primer specificity. Experiments were performed in triplicate, and results were normalized to GAPDH expression (2 ΔΔCT method).

### ELISA

Hearts were weighed and homogenized in ice-cold physiological saline at a 1:9 ratio. The homogenate was then centrifuged at 3000 rpm at 4 °C for 10 min, and the supernatant was collected for analysis. Total protein was quantified via Coomassie blue staining. The concentrations of types I and III collagen and PGE2 in the cell culture and heart tissue were detected by ELISA following the manufacturer’s instructions (R&D).

### Statistical analysis

Data are presented as the mean ± SD and were analyzed using SPSS 17.0 and GraphPad Prism 6. Student’s *t* test or one-way analysis of variance (ANOVA) was applied to compare the differences between experimental groups. *P* < 0.05 was considered statistically significant.

## Results

### Characteristics of the diabetic cardiomyopathy rat model

To estimate the DCM rat model, daily food and water intake of rats, fasting blood glucose (FBG), serum insulin (INS), total cholesterol (TC), triglyceride (TG) in the serum, and indicators reflecting cardiac diastolic and contractile functions, including LVEF, FS, and E/A ratio of echocardiography were determined at 12 weeks. Daily food and water intake of DCM rats were obviously higher than those of normal rats (Supplemental Fig. 1A, B). FBG, INS, TC, and TG in DCM rat serum were markedly increased compared to normal rats (Supplemental Fig. 1C–F). Moreover, echocardiogram results showed that LVEF, FS, and E/A ratio of DCM rats were decreased significantly compared to normal rats (Supplemental Fig. 1G–I). The above results reflected that basic metabolism, glucose and lipid metabolism, and cardiac function of DCM rats were injured and abnormal.

### Identification of MSC and cardiac fibroblast characteristics

To identify MSC characteristics, microscopy and flow cytometry were used to observe cell morphology and phenotype. MSCs were presented as the spindle shape and aggregated like swirls under the microscope. More than 99% of Passage 3 MSCs were positive for CD29 and CD90 but negative for CD34, CD45, and CD11b by flow cytometry (Supplemental Fig. 2A). In addition, Oil Red O and Alizarin Red staining showed that MSCs have differentiated to adipocytes and osteoblasts successfully (Supplemental Fig. 2B, C).

To identify fibroblast characteristics, cardiac fibroblasts were determined by microscopy and immunofluorescence. Fibroblasts were presented as multi-spindle-like or star-shaped flat cells under the microscope. Immunofluorescence showed that fibroblasts were positive for vimentin and negative for cTnT (Supplemental Fig. 2D).

To investigate the fate of MSCs in vivo, luciferase-labeled MSCs were intravenously injected into rats and tracked their distribution in vivo by optical bioluminescence imaging at 1 day, 1 week, and 2 weeks, respectively. The results showed that the labeled MSCs were enriched mainly in the lungs after the 1-day infusion. And the quantification of labeled cells was decreased more than 50% of the amount at 1 week, left about ~ 1/8 at 2 weeks from in vivo and ex vivo. However, other organs including heart hardly had labeled MSCs from our results (Supplemental Fig. 3A).

### MSCs ameliorated glucose, lipid metabolism, and cardiac function in DCM rats

**Fig. 1:**
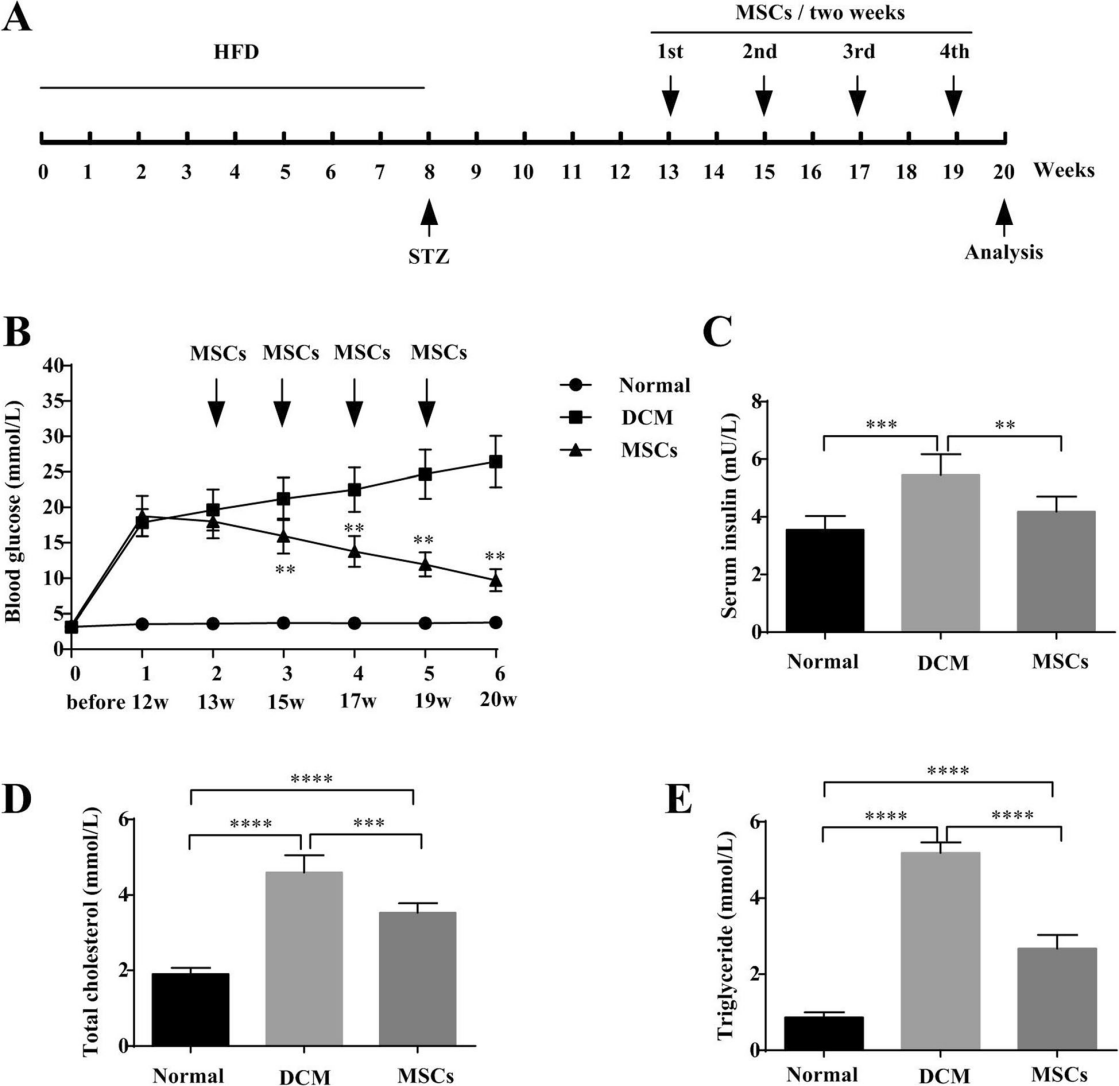
MSCs improved glucose and lipid metabolism of DCM rats. DCM rats were fed high-fat diet (HFD) for 8 weeks and intraperitoneally injected with a single dose of 25 mg/kg STZ. After 4 weeks’ observation and determination, MSCs were infused into DCM rats via tail vein every 2 weeks and 4 times in total (**a**). During the research, fasting blood glucose was detected at the beginning, 12 weeks, within 48 h after every MSC infusion, and at 20 weeks (**b**). *n* = 6, **P < 0.005 vs. DCM group. At the end of the experiment, serum insulin (**c**), total cholesterol (**d**), and triglyceride (**e**) in normal, DCM, and MSC-treated rats were determined, respectively. *n* = 6, **P < 0.005, ***P < 0.0005, ****P < 0.0001

**Fig. 2:**
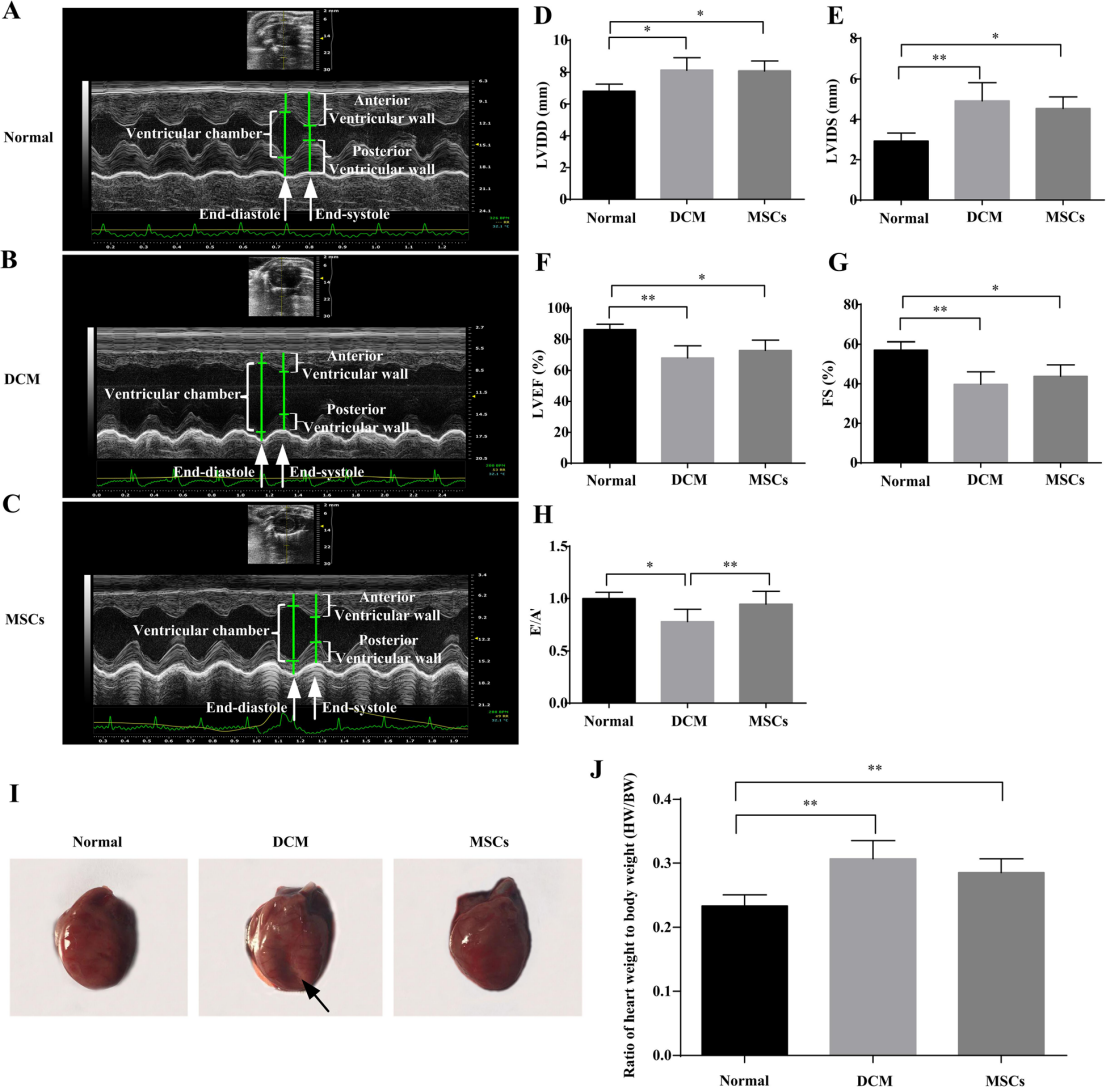
MSCs ameliorated abnormal cardiac structure and function in DCM rats. After MSC treatment, echocardiography (**a–c**) was used to evaluate cardiac structure, including left ventricular end-diastolic internal diameters (LVIDD**; d**) and LV end-systolic internal diameters **(**LVIDS**; e**), cardiac systolic function containing left ventricular ejection fraction **(**LVEF**; f**) and fractional shortening (FS**; g**) and cardiac diastolic function, including the early to late diastolic mitral annular velocity (E’/A’**; h**) ratio for different groups. *n* = 3–5, *P < 0.05, **P < 0.005. Rat cardiac photographs were taken to present the whole heart morphology of different groups after rats were euthanized (**i**). At the end of the experiment, body weight and heart weight of rats were measured, and the ratio of heart weight to body weight (HW/BW) was calculated (**j**). *n* = 6, **P < 0.005. **a**–**c** The size of ventricular chamber and the thickness of the anterior and posterior ventricular wall at the end-diastole and end-systole, respectively. Arrow in **i** indicated the collapsed ventricular wall

To evaluate the effect of MSCs on DCM rats, serological parameters including FBG, INS, TC, and TG, ratio of heart weight to body weight (HW/BW), and echocardiography indicators including LVIDD, LVIDS, LVEF, FS, and E’/A’ were performed after 4 MSCs infusions to reflect glucose and lipid metabolism, cardiac structure and function (Fig. 1a). Serological results showed that FBG, INS, TC, and TG in MSC-treated rats were markedly decreased compared to DCM rats (Fig. 1b–e). Moreover, echocardiogram results indicated that LVIDD and LVIDS of DCM hearts were obviously larger than those of normal hearts, while MSC infusion decreased LVIDD and LVIDS compared to DCM rats (Fig. 2a–e). Contractile function indexes including LVEF and FS, and diastolic function index E’/A’ in DCM rats were dramatically decreased compared to those in normal rats. However, LVEF and FS were numerically increased, while E’/A’ was markedly increased after MSC treatment (Fig. 2f–h). Consistent with echocardiogram results, the morphology of the whole heart showed that DCM hearts had collapsed ventricular walls, and these phenomena were alleviated after MSC treatment (Fig. 2i). Additionally, the HW/BW of DCM rats was significantly larger than that of normal rats, whereas HW/BW was numerically decreased after MSC infusion (Fig. 2j). These data demonstrated that MSC infusion might improve abnormal glucose and lipid metabolism of DCM rats. MSCs might also ameliorate dilated hearts and abnormal cardiac contractile and diastolic function in DCM rats.

### MSC infusion ameliorated myocardial fibrosis in DCM rats

**Fig. 3:**
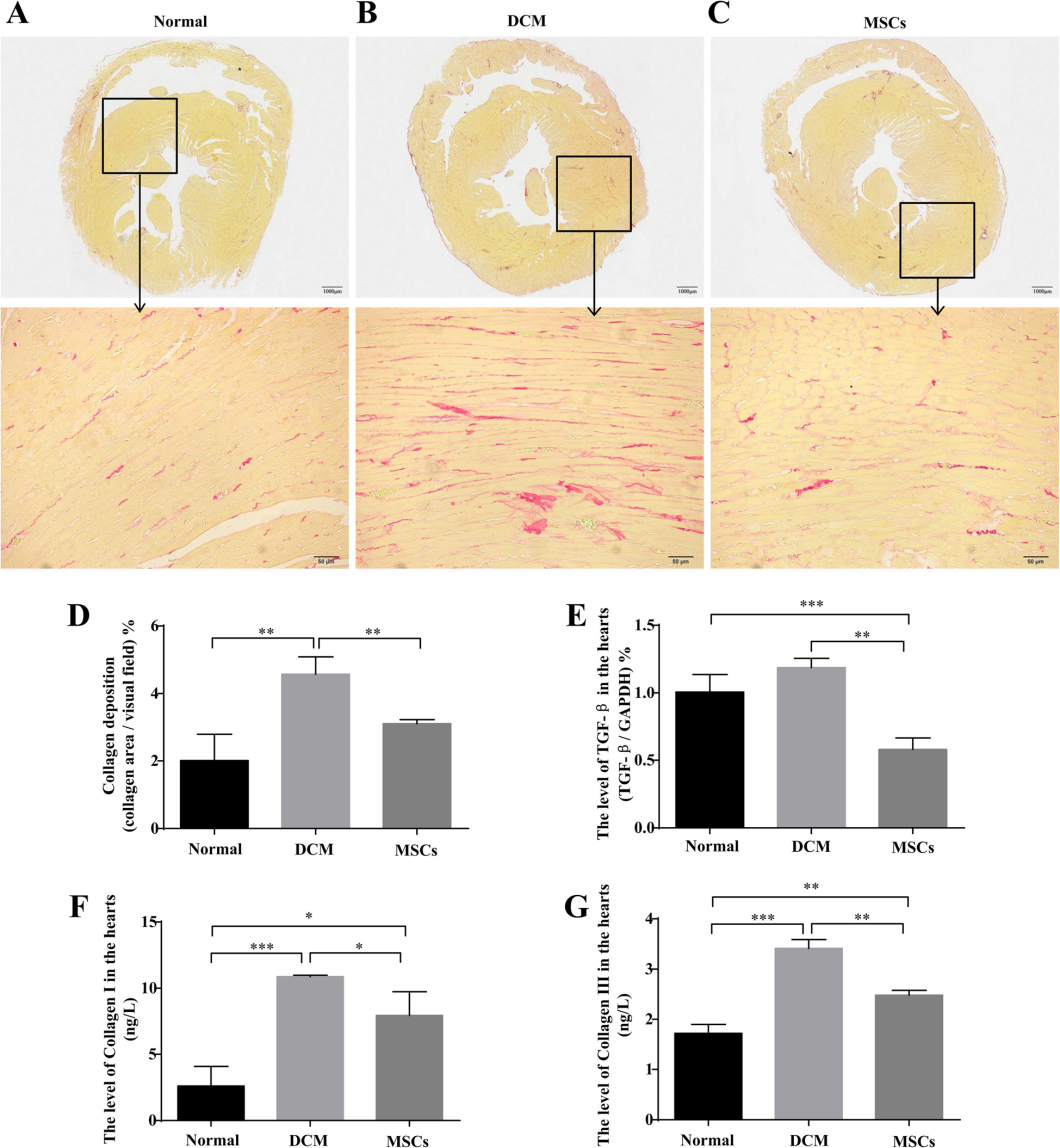
MSC infusion ameliorated myocardial fibrosis in DCM rats. At the end of the experiment, Sirius Red staining (**a–c**) for the hearts of three groups was performed, and quantification of the collagen deposition (**d**) was also calculated to reflect the degree of myocardial fibrosis. Moreover, the mRNA levels of TGF-β in the hearts were detected by PCR (**e**), and the protein levels of Collagen I (**f**) and Collagen III (**g**) in the hearts were determined by ELISA assay. *n* = 3, Scale bar = 1000 μm (upper), 50 μm (lower), *P < 0.05, **P < 0.005, ***P < 0.0005. Inset and arrow show partially enlarged area

To assess the effect of MSCs on myocardial fibrosis amelioration, Sirius Red staining, mRNA level of TGF-β, and protein levels of Collagen I and Collagen III in DCM hearts were performed to reflect the degree of collagen deposition at week 20. As shown in Fig. 3a–c and d, Sirius Red staining and quantification of myocardial fibrosis results displayed that DCM hearts had obvious collagen deposition, whereas MSC infusion markedly reduced the collagen deposition in DCM hearts. Moreover, the mRNA level of TGF-β increased in DCM hearts but decreased markedly after MSC treatment (Fig. 3e). In addition, the protein levels of Collagen I and Collagen III detected by ELISA were found to be dramatically increased in DCM hearts, whereas they were decreased in MSC-treated hearts (Fig. 3f–g). These data demonstrated that DCM hearts had collagen accumulation and deposition, and MSCs infusion might alleviate myocardial fibrosis.

### MSCs ameliorated cardiac dysfunction and fibrosis of DCM rats partially via PGE2

According to our research and previous studies, MSCs might secrete cytokines containing PGE2 to exert regulatory effects. To investigate whether MSCs-released PGE2 might affect on myocardial fibrosis and cardiac dysfunction of DCM, we injected purified PGE2-deficient MSCs into DCM rats (Supplemental Fig. 3B). The levels of PGE2 were obviously decreased in PGE2-deficient MSCs than those in non-treated MSCs and MSCs with control lentivirus (Supplemental Fig. 3C). From our results, LVAWS, LVEF, and FS of the MSC-PGE2^−/−^ group were significantly lower than those of MSCs-treated group (Supplemental Fig. 4). Otherwise, the collagen deposition and the levels of Collagen I, III in the hearts of MSC-PGE2^−/−^ group were markedly increased compared to MSC-treated group (Supplemental Fig. 5A–D), while there were no statistical differences of the blood glucose between MSC and MSC-PGE2^−/−^ groups (Supplemental Fig. 5E). The above data demonstrated that MSCs might ameliorate cardiac dysfunction and fibrosis of DCM via the secretion of PGE2.

### MSCs alleviated fibroblasts proliferation and collagen synthesis in vitro via the COX-2-PGE2 pathway

**Fig. 4:**
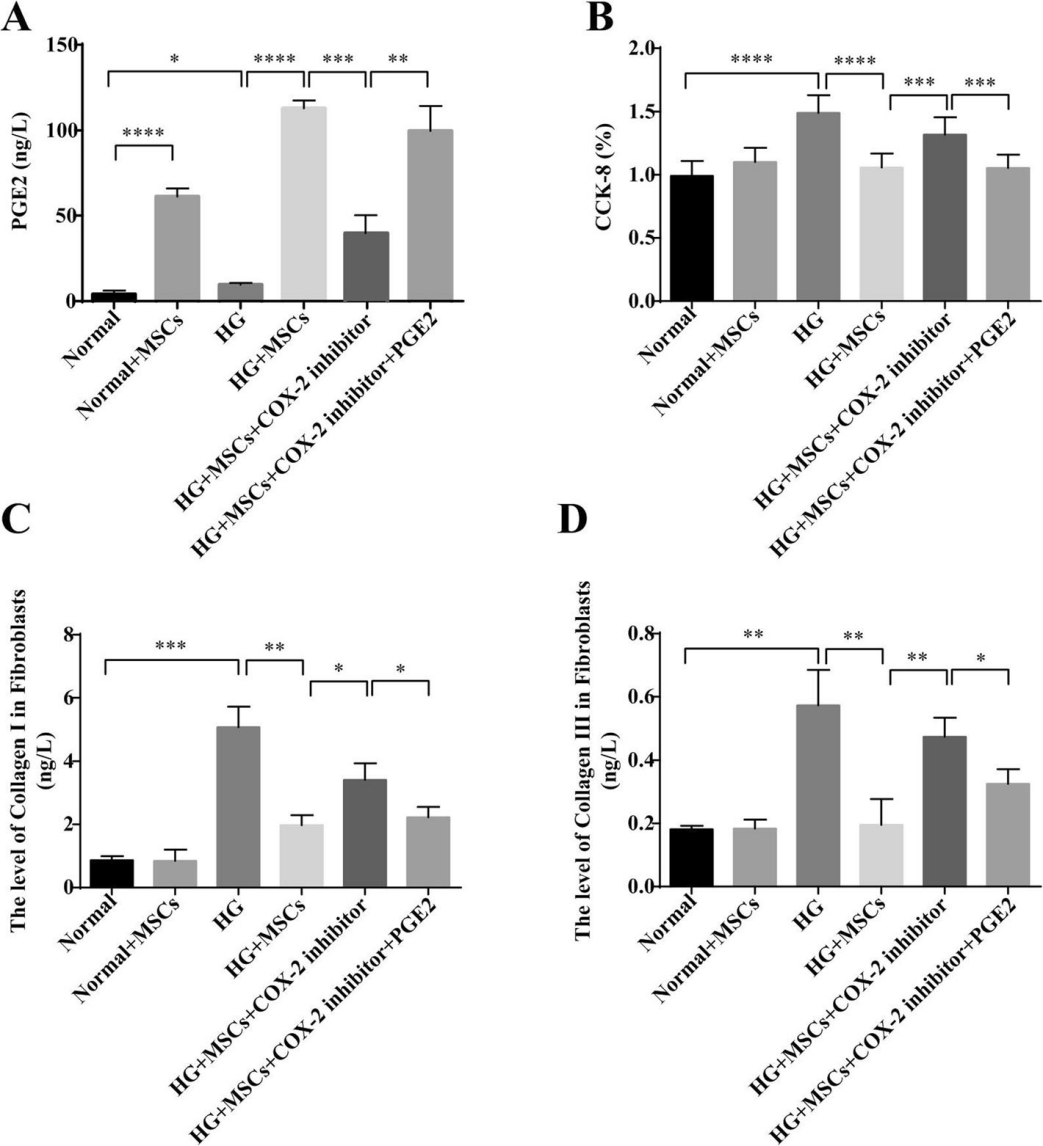
MSCs alleviated fibroblast proliferation and collagen synthesis in vitro via the COX-2-PGE2 pathway. To further explore the underlying mechanism, cardiac fibroblasts were induced by high glucose and cocultured with MSCs in vitro. Otherwise, MSCs were pretreated with COX-2 inhibitor adding PGE2 or not. The protein level of PGE2 in the supernatant was measured by ELISA (**a**). Cardiac fibroblasts growth rate was evaluated by CCK-8 assay to reflect fibroblast proliferation (**b**). Additionally, the protein levels of Collagen I (**c**) and Collagen III (**d**) in the supernatant were determined by ELISA to reflect the degree of collagen synthesis. *n* = 3, *P < 0.05, **P < 0.005, ***P < 0.0005, ****P < 0.0001

To further explore the underlying mechanism of MSCs on myocardial fibrosis amelioration via decreasing collagen synthesis, we treated cardiac fibroblasts with high glucose (HG) in vitro to mimic the DCM microenvironment in vivo, then co-cultured fibroblasts with MSCs. We found that the MSC-treated group in HG conditions had high levels of PGE2 (Fig. 4a). Therefore, we used a COX-2 inhibitor to inhibit PGE2 secretion. Consistently, the PGE2 level decreased in MSCs pre-treated with COX-2 inhibitor group (Fig. 4a). Based on these results, we further hypothesized that PGE2 might be involved in MSCs-associated fibrotic alleviation.

Moreover, CCK-8 assay and ELISA were used to detect the ability of cardiac fibroblast proliferation and collagen synthesis. CCK-8 assay showed that HG increased the growth rate of fibroblasts and MSCs coculturing decreased fibroblasts proliferation induced by HG (Fig. 4b). ELISA assay results showed that HG increased Collagen I and III levels, while the levels were decreased markedly after MSC treatment (Fig. 4c–d). These data indicated that HG increased cardiac fibroblast proliferation and collagen synthesis, but MSC treatment ameliorated this phenomenon.

To further demonstrate the underlying mechanism, we used MSCs pretreated with COX-2 inhibitor to inhibit the secretion of PGE2, and added PGE2 to this inhibited group. Notably, fibroblast proliferation in this inhibitor group showed increased cell growth rate compared to MSCs-treated group (Fig. 4b), and the protein levels of Collagen I and III also increased after adding COX-2 inhibitor (Fig. 4c, d). In contrast, both the fibroblast growth rate and the collagen synthesis were decreased again after adding PGE2 to the inhibition system (Fig. 4b–d). The above data indicated that the high level of PGE2 secreted from MSCs might be inhibited by COX-2 inhibitor, and the ability of MSCs to suppress fibroblasts proliferation and collagen synthesis might also be inhibited by COX-2 inhibitor. MSCs might ameliorate fibrosis by suppressing fibroblast proliferation and collagen synthesis via the COX-2-PGE2 pathway.

## Discussion

Diabetic cardiomyopathy (DCM) is a severe and advanced diabetic cardiac complication, which leads to high morbidity and mortality. DCM-related cardiac fibrosis results in pathological fibrosis, which is mediated by enhanced expression of pro-fibrotic factors like TGF-β and extracellular matrix (ECM) components such as collagen I and III, decreased extracellular matrix degradation, and cardiac dysfunction, which finally causes irreversible heart failure and even death [22–25]. The current clinical treatment for DCM relies on the improvement of heart failure symptoms. There is still no effective method to prevent the progression of myocardial fibrosis in DCM.

As a potential cell-based therapy, mesenchymal stem cell (MSC) administration has been considered as an alternative to conventional treatments [26]. Recently, more multiple studies have reported the efficiency of MSCs in the treatment of metabolic diseases, such as diabetes and cardiovascular diseases due to their immunomodulatory, anti-inflammatory, immune-privileged properties, and multilineage potential [26, 27]. In our previous studies, MSC infusion was able to decrease blood glucose, insulin resistance, and inflammation, and ameliorate the prognosis of type 2 diabetes efficiently [13, 28]. Many reports have demonstrated that MSCs might alleviate inflammation and improve cardiac function in myocardial infarction, while other studies have demonstrated the effects of MSCs on dilated cardiomyopathy and diabetic cardiomyopathy. However, only a few studies have discussed the potential mechanisms underlying the anti-fibrotic or anti-inflammatory effects of MSCs on myocardial fibrosis in DCM. Therefore, we carried out this study to explore the mechanism underlying the effect of MSCs on DCM.

Therefore, we established a DCM rat model that was induced by HFD combined with a low dose of STZ. Then MSC infusion was administrated four times to observe the ability of MSCs to ameliorate cardiac function and structure. In the present study, DCM rats showed abnormal basal metabolism of glucose and lipids, and impaired cardiac structure and function compared to those of normal rats. However, our research showed that MSC treatment could alleviate glucose and lipid metabolic injury in DCM rats. Moreover, the dilated ventricular chamber and collapsed wall showed improvement in the MSC-treated hearts as seen in echocardiogram and heart photogram. The HW/BW ratio was partially ameliorated compared to that of DCM rats. In addition, consistent with previous reports, cardiac diastolic function improved significantly, while the contractile function was partially ameliorated after MSC infusion. Thus, our research demonstrated that MSC treatment might alleviate basal metabolism and abnormal cardiac structure and function in DCM rats.

To specifically determine the degree and peculiarity of damaged DCM heart, we performed histopathological staining of heart tissue and detected the expression of fibrotic factors to assess cardiac fibrosis. In particular, DCM hearts showed significant collagen deposition, whereas MSC-treated hearts showed decreased collagen deposition compared to DCM hearts. Consistent with this observation, the indicators such as TGF-β, collagen I, and collagen III which reflect the degree of fibrosis were significantly enhanced in DCM hearts compared to those in normal hearts. These targets were markedly ameliorated after MSC treatment. Consistent with previous research, we demonstrate that MSCs can improve the degree of myocardial fibrosis in DCM hearts.

Importantly, to further explain the mechanism of alleviation of cardiac fibrosis by MSCs, we induced cardiac fibroblasts with HG to mimic the DCM microenvironment in vitro. Then, MSCs were cocultured with the induced fibroblasts to study the effect on fibrosis. We detected fibrosis in vitro by measuring fibroblast proliferation and collagen secretion by fibroblasts with CCK-8 assay and ELISA, respectively. The results indicated that cardiac fibroblasts induced with HG had significantly increased growth rate and collagen secretion compared to the normal group. However, after coculturing with MSCs, the fibroblast proliferation and collagen secretion were markedly decreased, demonstrating that MSCs might alleviate the degree of fibrosis by decreasing fibroblast proliferation and collagen production.

However, little is known about the mechanism of the effect of MSCs on myocardial fibrosis in DCM hearts. MSCs usually exert an immunomodulatory effect by secreting cytokines, such as PGE2 [29, 30]. MSCs could secrete certain cytokines to trigger paracrine and exocrine responses, which further exert systemic influence. Similarly, optical bioluminescence imaging results in our research showed that intravenous MSCs rarely distributed to tissues other than lungs, but might exert systemic regulatory effects through their secreted factors. Until now, only a few studies have explored the mechanistic details regarding the role of MSCs in DCM hearts. Thus, we aimed to explore the possible underlying mechanism of the effect of MSCs on cardiac fibrosis and dysfunction of DCM in vivo and cardiac fibroblasts in HG culture conditions in vitro. Firstly, according to our previous studies and other similar reports [13, 30], we observed that PGE2 was secreted by MSCs during inflammation and immune response. Secondly, several recent studies on pulmonary and intestinal fibrosis have mentioned the anti-fibrotic effect of PGE2. In addition, several studies reported the anti-inflammatory and anti-fibrotic properties of PGE2 in cardiac fibrosis [15, 16, 31]. Therefore, we chose PGE2 as our target cytokine and further studied the association between the effects of MSCs on cardiac fibrosis and the PGE2-associated pathway. Our research used PGE2-deficient MSCs to observe the effect of MSCs-secreted PGE2 on DCM and determined that the level of PGE2 in the PGE2-deficient MSCs was markedly lower than that in non-treated or control-lentivirus MSCs. From our results, rats in MSC-PGE2^−/−^ group had obviously decreased cardiac function and increased collagen production and deposition in their hearts than the MSCs-treated rats. However, PGE2-deficient MSCs infusion had less effect on blood glucose in DCM rats. Thus, MSCs could exert the anti-fibrotic effect on DCM via secreting PGE2.

Moreover, it was observed that the concentration of PGE2 in MSC-treated supernatant was significantly higher than that in the non-treated group in vitro, which strengthened our determination to study the PGE2 pathway. PGE2 is synthesized through arachidonic acid metabolism with COX-2 being the key enzyme [32]. Thus, we used a COX-2 inhibitor to suppress the expression of PGE2. In line with our expectations, COX-2 inhibitor markedly reduced PGE2 levels. In addition, the ability of MSCs to prevent fibroblast proliferation and collagen secretion was influenced by the COX-2 inhibitor. To further determine the effect of PGE2, we added PGE2 to this inhibition group. As expected, the concentration of PGE2 and the abilities of fibroblasts proliferation and collagen secretion changed inversely compared to the inhibition group. To summarize, we demonstrated the role of the COX-2-PGE2 pathway in the anti-fibrotic effect of MSCs under HG conditions.

## Conclusion

**Fig. 5:**
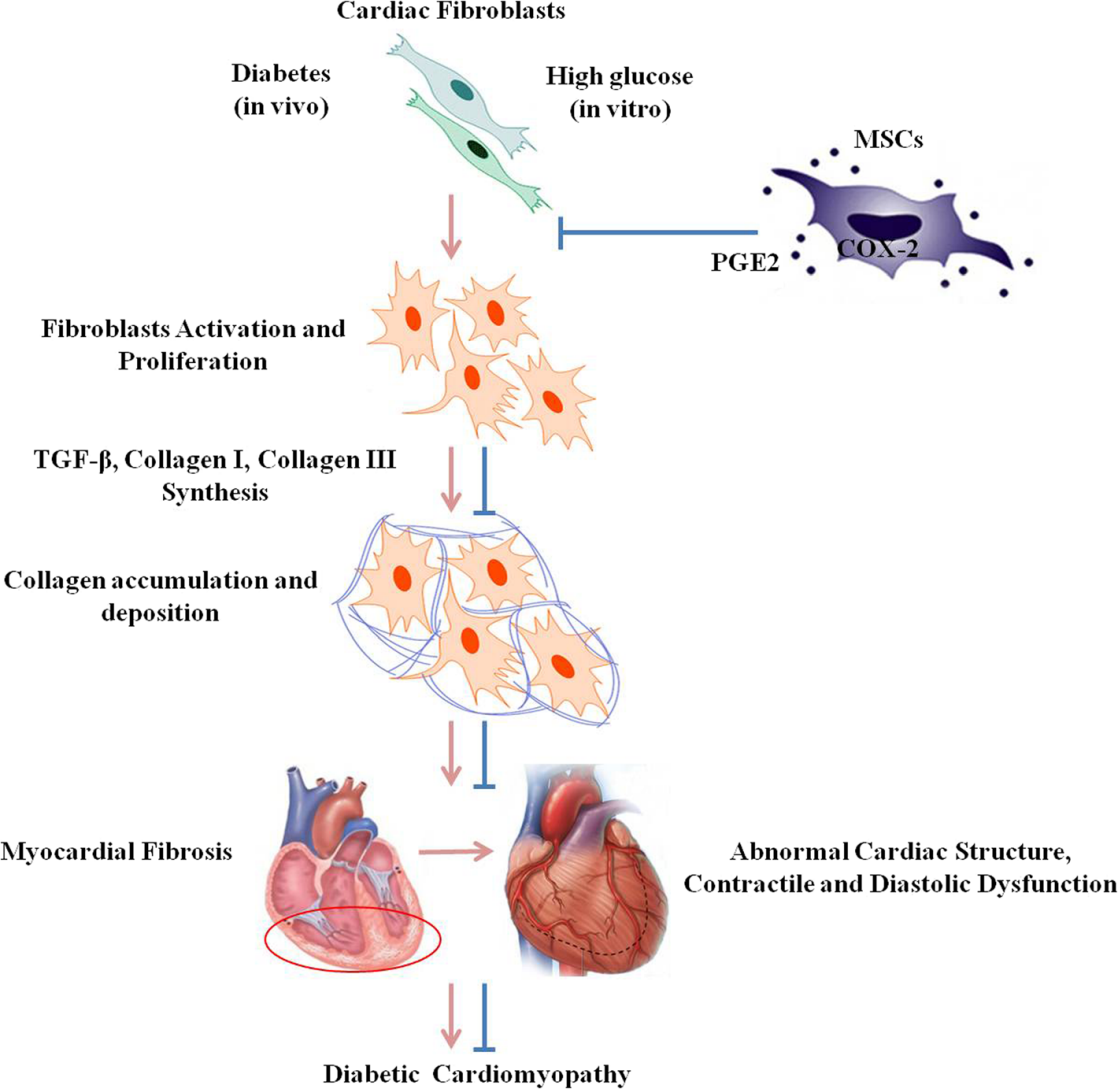
Graphic abstract. Long-term diabetes status (in vivo) and high-glucose microenvironment (in vitro) led to the activation and proliferation of cardiac fibroblasts and subsequently increased the synthesis and secretion of TGF-β, Collagen I and Collagen III, triggering the collagen accumulation and deposition among cardiomyocytes and around vessels, which cause myocardial fibrosis. Lasting cardiac fibrosis without effective intervention induced irreversible injury of cardiac structure and function, resulting in diabetic cardiomyopathy. However, adipose-derived MSC infusion could ameliorate the proliferation and collagen secretion of cardiac fibroblasts caused by high glucose. The underlying mechanism might involve the COX-2 enzyme-mediated arachidonic acid metabolism to release cytokine PGE2 by MSCs under a high-glucose microenvironment. Consequently, MSCs might alleviate myocardial fibrosis and ameliorate abnormal cardiac structure and function of DCM via the COX-2-PGE2 pathway

In general, MSC infusion might ameliorate metabolism and alleviate heart injury and fibrosis. Specifically, MSCs might suppress cardiac fibroblast proliferation and its collagen secretion under HG conditions in vitro. Above all, our current research clarified the positive effect of MSCs on myocardial fibrosis in DCM, and first demonstrated that the MSCs-related COX-2-PGE2 pathway might be the underlying mechanism in vivo and in vitro preliminarily (Fig. 5). On this basis, our study might provide a novel and efficient method for preventing the development of DCM.

## Supplementary information

**Additional files 1.** Supplemental Figure 1. The establishment and characteristics of DCM rat model.

**Additional file 2.** Supplemental Figure2. Identification of AD-MSC and cardiac fibroblast characteristics.

**Additional file 3.** Supplemental Figure3. In vivo distribution of MSC and identification of PGE2-deficient MSC.

**Additional file 4.** Supplemental Figure4. MSC ameliorated abnormal cardiac structure and function of DCM rats via PGE2.

**Additional file 5.** Supplemental Figure5. MSC infusion ameliorated myocardial fibrosis in DCM rats partially via PGE2.

**Additional file 6.** Supplemental Table 1. Primer sequences for PCR.

## Data Availability

Data and study materials are available.

## Abbreviations

(AD)-MSCs: (adipose-derived) mesenchymal stem cells
CCK-8: Cell counting kit-8 assay
COX-2: Cyclooxygenase-2
DCM: Diabetic cardiomyopathy
E/A: The ratio of early to late diastolic transmitral flow velocity
E’/A’: The ratio of early to late diastolic mitral annular velocity
ELISA: Enzyme-linked immunosorbent assay
FBG: Fasting blood glucose
FBS: Fetal bovine serum
FS: Fractional shortening
HFD: High-fat diet
HG: High glucose
HW/BW: The ratio of heart weight to body weight
INS: Serum insulin
LVAWS: Left ventricular end-systolic anterior wall
LVEF: Left ventricular ejection fraction
LVIDD: Left ventricular end-diastolic internal diameters
LVIDS: Left ventricular end-systolic internal diameters
LVPWS: Left ventricular end-systolic posterior wall thickness
PCR: Polymerase chain reaction
PGE2: Prostaglandin E2
STZ: Streptozotocin
TC: Total cholesterol
TG: Triglyceride
TGF-β: Transforming growth factor-β
