# Supplementary figures and images for: Mesenchymal stem cells ameliorate myocardial fibrosis in diabetic cardiomyopathy via the secretion of prostaglandin E2
Source: Stem Cell Res Ther. 2020 Mar 17;11:122. doi: 10.1186/s13287-020-01633-7 (PMC7079514; doi:10.1186/s13287-020-01633-7)

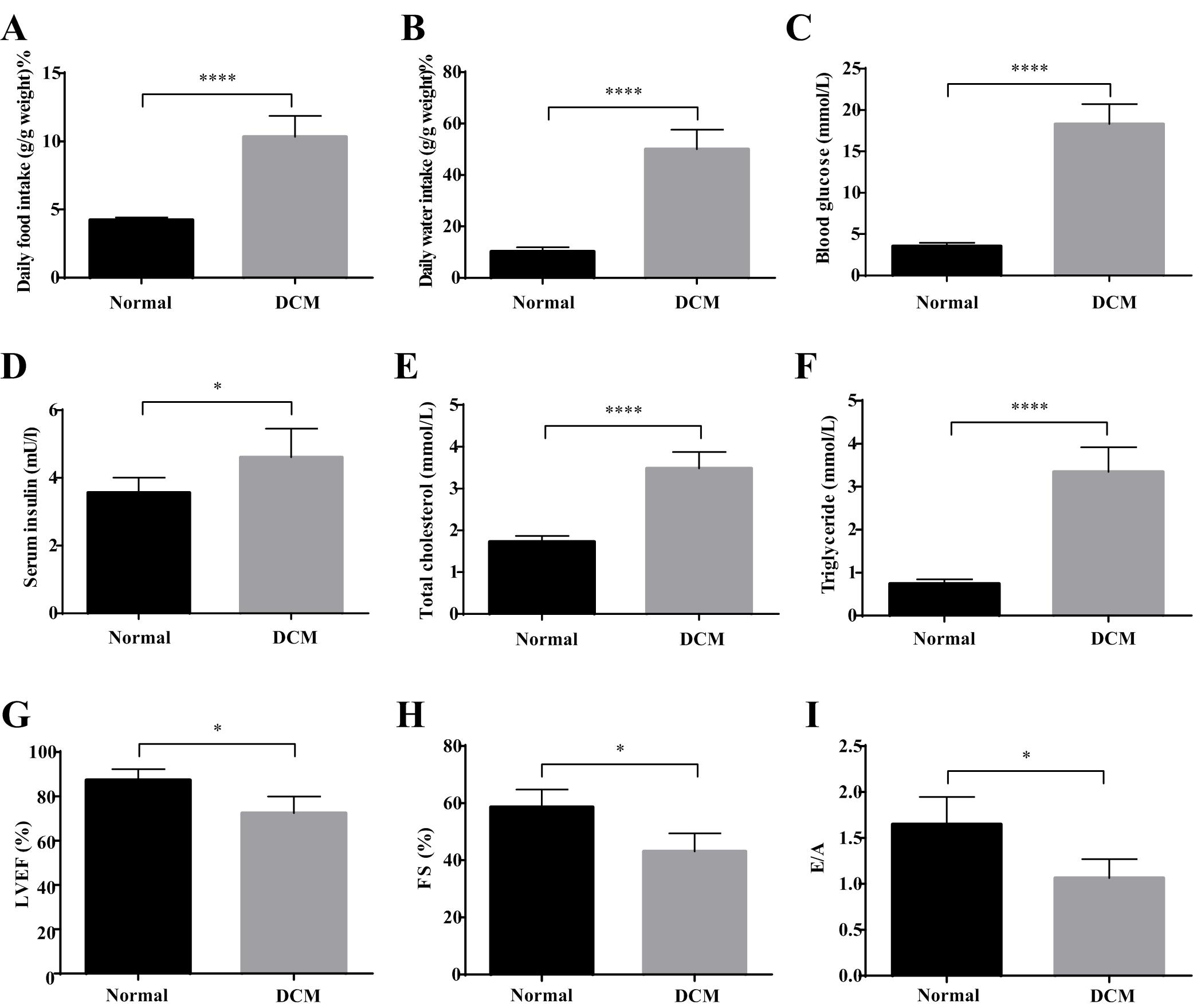

Supplement: Supplementary file 1 — Additional files 1. Supplemental Figure 1. The establishment and characteristics of DCM rat model. [file 13287_2020_1633_MOESM1_ESM.jpg]

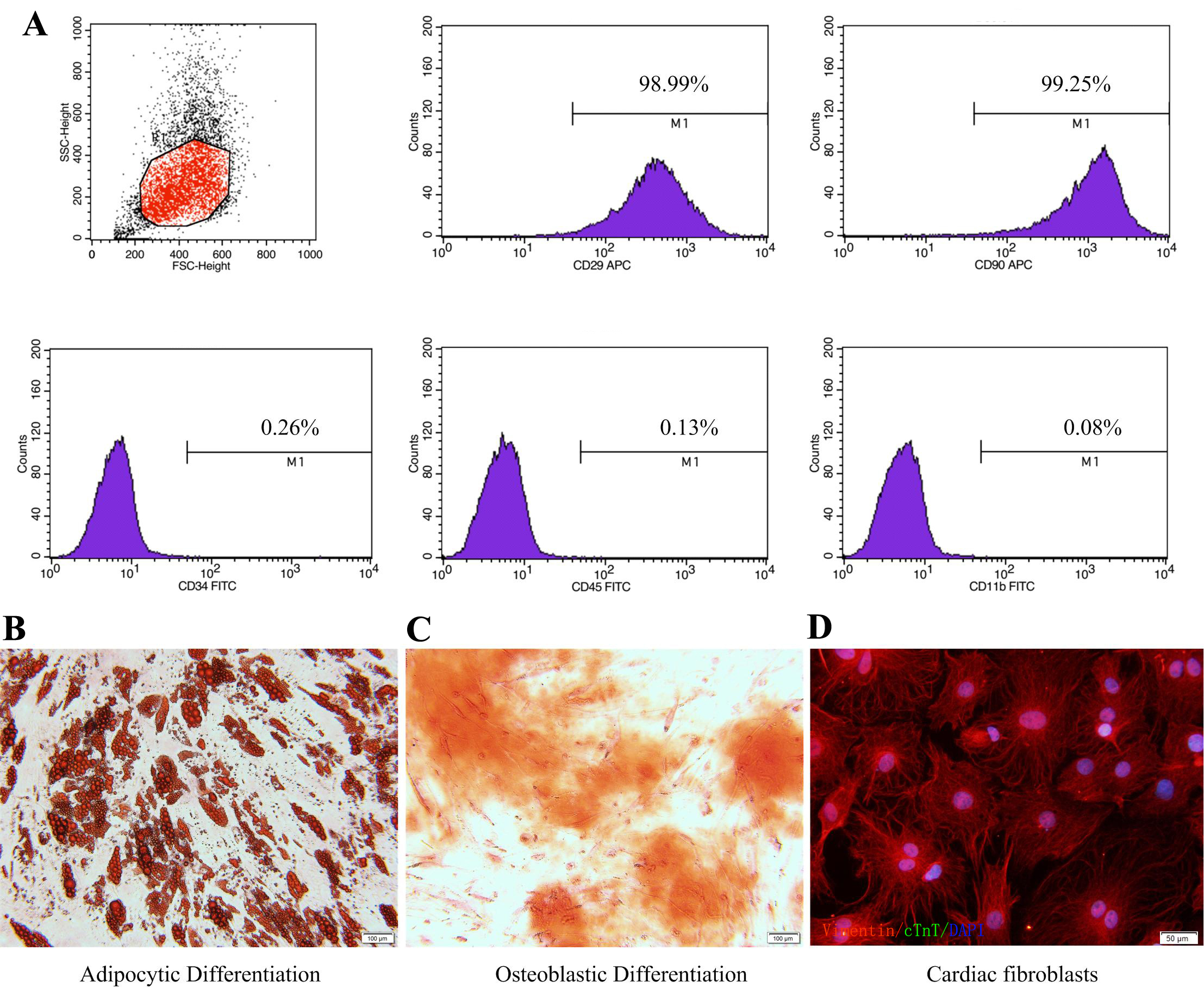

Supplement: Supplementary file 2 — Additional file 2. Supplemental Figure2. Identification of AD-MSC and cardiac fibroblast characteristics. [file 13287_2020_1633_MOESM2_ESM.jpg]

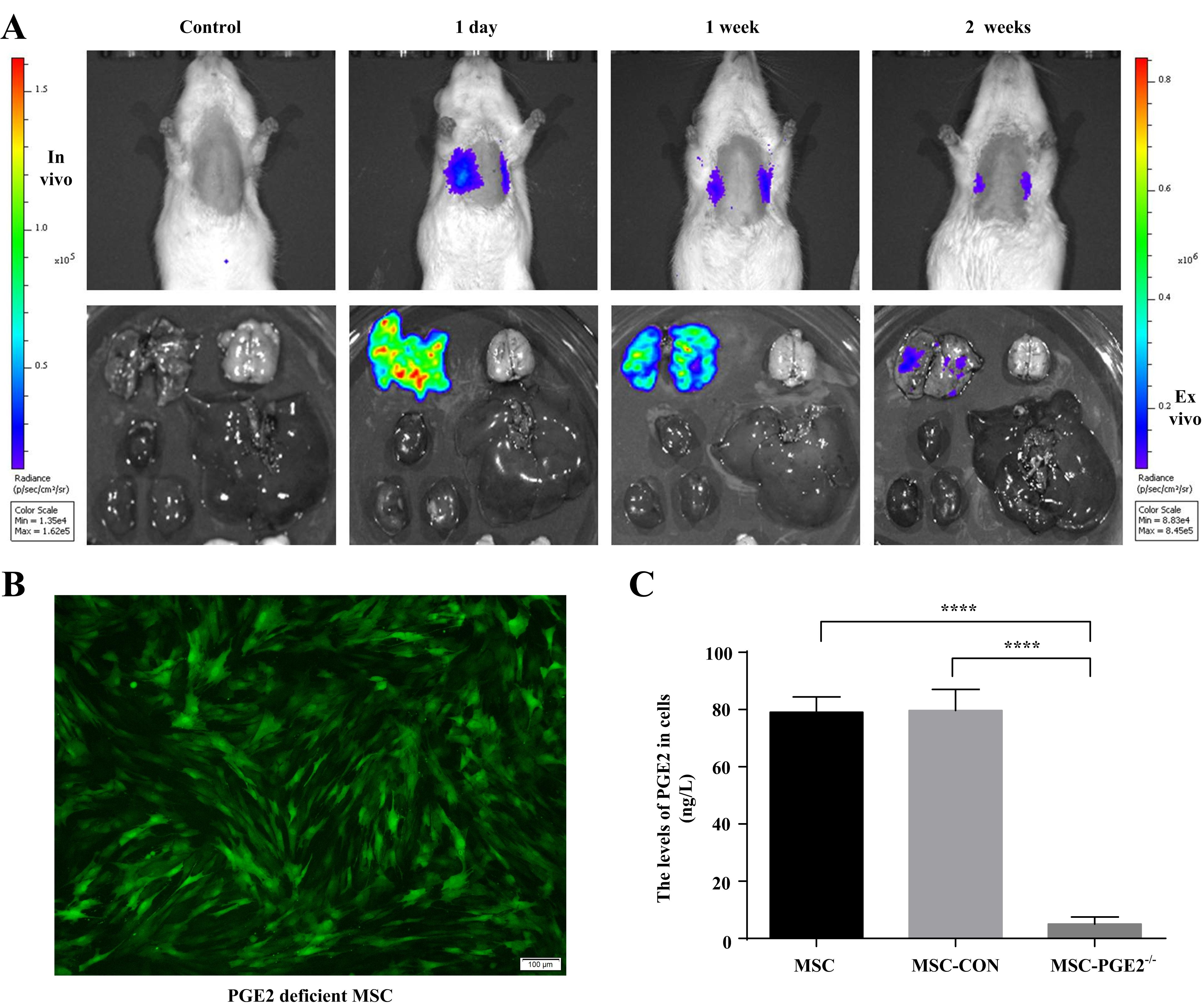

Supplement: Supplementary file 3 — Additional file 3. Supplemental Figure3. In vivo distribution of MSC and identification of PGE2-deficient MSC. [file 13287_2020_1633_MOESM3_ESM.jpg]

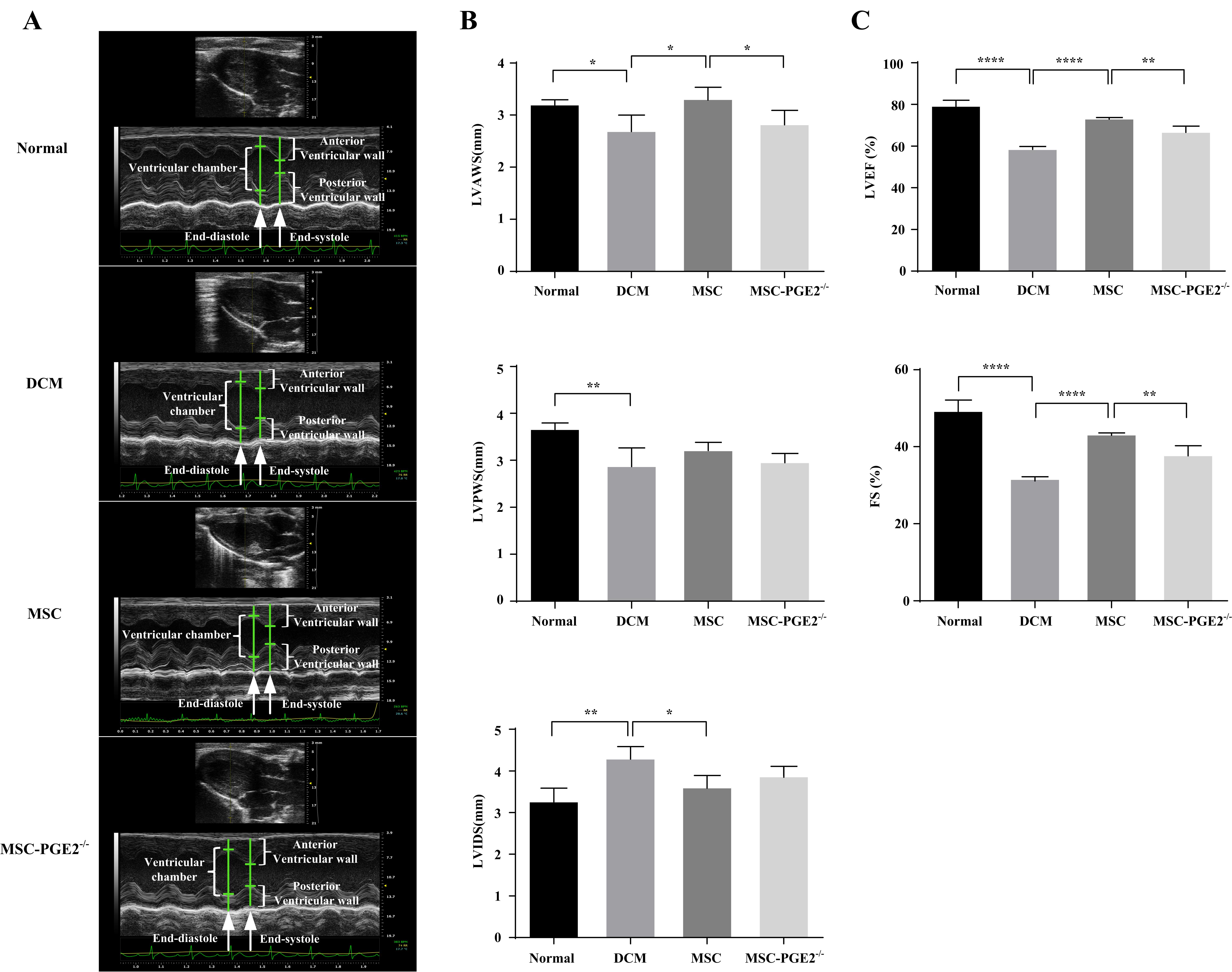

Supplement: Supplementary file 4 — Additional file 4. Supplemental Figure4. MSC ameliorated abnormal cardiac structure and function of DCM rats via PGE2. [file 13287_2020_1633_MOESM4_ESM.jpg]

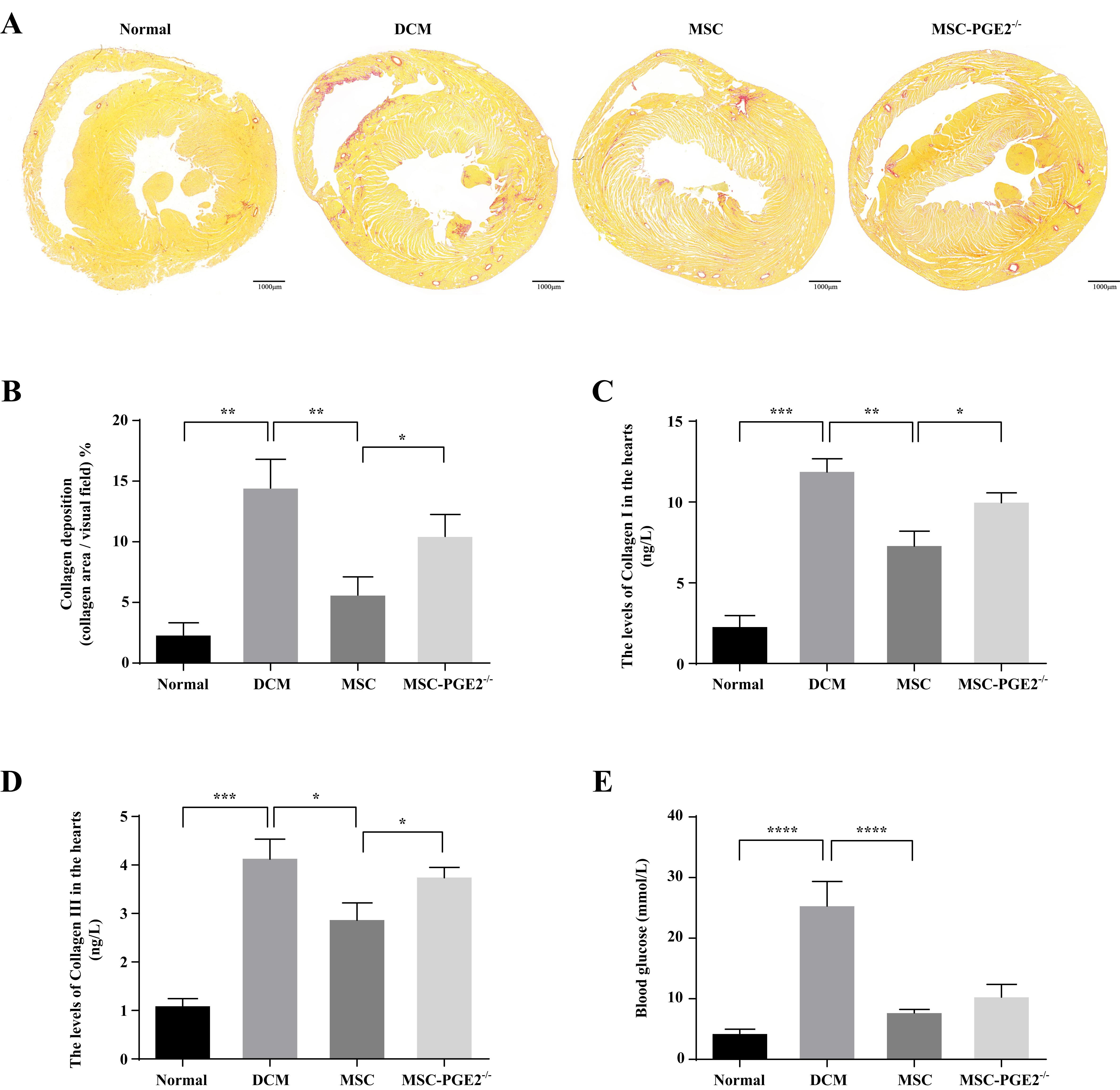

Supplement: Supplementary file 5 — Additional file 5. Supplemental Figure5. MSC infusion ameliorated myocardial fibrosis in DCM rats partially via PGE2. [file 13287_2020_1633_MOESM5_ESM.jpg]
