# Supplementary material for: Mesenchymal stem cells ameliorate myocardial fibrosis in diabetic cardiomyopathy via the secretion of prostaglandin E2
Source: Stem Cell Res Ther. 2020 Mar 17;11:122. doi: 10.1186/s13287-020-01633-7 (PMC7079514; doi:10.1186/s13287-020-01633-7)
Supplement: Supplementary file 6 — Additional file 6. Supplemental Table 1. Primer sequences for PCR. [file 13287_2020_1633_MOESM6_ESM.docx]

Supplemental Table 1. Primer sequences for PCR

| Target gene | Primers sequences | | Annealing T (℃) |
| --- | --- | --- | --- |
| GAPDH | F | 5’- GGCAAGTTCAACGGCACAG - 3’ | 60 |
|  | R | 5’- CGCCAGTAGACTCCACGACA - 3’ | 60 |
| TGF-β | F | 5’- ATAGCAACAATTCCTGGCGTTACCTT- 3’ | 60 |
|  | R | 5’- CCTGTATTCCGTCTCCTTGGTTCAG - 3’ | 60 |
